# Moral or Dirty Leadership: A Qualitative Study on How Juniors Are Managed in Dutch Consultancies

**DOI:** 10.3390/ijerph15112506

**Published:** 2018-11-09

**Authors:** Onno Bouwmeester, Tessa Elisabeth Kok

**Affiliations:** 1 School of Business and Economics, Vrije Universiteit Amsterdam, 1081 HV Amsterdam, The Netherlands; 2 PwC Amsterdam, 1066 JR Amsterdam, The Netherlands; tessa.kok@hotmail.com

**Keywords:** work organization, dirty work, moral leadership, taint normalization, management consulting

## Abstract

Professional service firms in Western Europe have a reputation for putting huge pressures on their junior employees, resulting in very long work hours, and as a consequence health risks. This study explores moral leadership as a possible response to the stigma of such dirty leadership. We conducted semi-structured interviews with 12 consultant managers and with each one of their juniors, and found that managers put several pressures on their juniors; these pressures bring high levels of stress, lowered wellbeing and burnout. Society considers such a pressuring leadership style morally dirty. To counteract the experience of being seen as morally dirty, we found that consultant managers were normalizing such criticisms as commonly assumed in dirty work literature. However, they also employed several moral leadership tactics to counteract the negative consequences criticized in society. However, in addition to the well-known individual-level tactics, consultant managers and their juniors also reported moral leadership support at the organizational level, like institutionalized performance talks after every project, trainings, specific criteria for hiring juniors, and policies to recognize and compliment high performance. Still, we cannot conclude these moral leadership approaches are moral by definition. They can be used in an instrumental way as well, to further push performance.

## 1. Introduction

*“Consulting overall is a stressful lifestyle. Travel does suck, and it doesn’t get any better. You’re at the demand of your manager.… at all times, and deadlines are seemingly impossible to meet”.*

*(www.wallstreetoasis.com, entry 2014)*

When Hughes introduced the concept of dirty work, he claimed that “dirty work of some kind is found in all occupations” [1] (p. 319). High-status professions are no exception. For instance, recently, bankers’ dirty image has been studied, due to their risky management style, lack of customer care and extreme bonus culture, leading to a financial crisis and public scandals [2,3]. Accountants are in the news as well for big accounting errors and they self-report shame for dirty tasks like providing “ritualized information” and producing “ignored documents”, which they consider “dirty work” [4] (p. 235). Popular criticisms also target consultants for their lack of expertise and overly high fees, lack of independence, and a focus on rationalization over human values [5,6]. Mostly consultants’ clients are identified as victims of such morally dirty practices [7,8,9]. Such public criticisms undermine the reputation of consultants and contribute to the occupation’s dirty image [10,11]. Despite the profession’s high status in general, society disapproves certain dirty aspects of the work, like “laying off” people in client organizations [12] (p. 599).

A different moral problem criticized in society is that managers in professional service industries like banking [13] and law firms [14,15] put quite strong pressures on their employees. The pressures go far beyond standards of social desirability, even to the extent of violating labour laws. The consulting industry, for instance, is known for burnout, mental problems, stress, and disturbed work–life balance due to demanding clients and managers [16,17,18,19,20,21]. As a consequence, manager criticisms abound in consultant jokes, cartoons and on Internet fora (see for instance managementconsulted.com or www.wallstreetoasis.com). Members and former members of the occupation point at the moral dirtiness of such pressuring leadership, and of the manager job.

The constructs of dirty work and occupational stigma have initially been developed in sociology by Goffman and Hughes. Society stigmatizes in particular low-status occupations like hangman or janitors, similar to groups like drunks or ex-convicts [22,23]. Ashforth et al. [24] have added a social psychological perspective. They have explored how dirty workers and their managers respond to the pressure of feeling stigmatized, and found in their empirical studies that dirty workers respond by normalizing the taint experience in order to protect their self-esteem, and to reduce the stress caused by the feeling of being stigmatized. They also found that managers were helping employees with normalizing the experience of stigma. Luyendijk [25] finds such a phenomenon of creating a “protective bubble” to be quite prominent in the banking industry.

However, whereas insiders are assumed to reduce feelings of stress caused by a critical public opinion [26], outsiders produce such stress for a reason. In case of moral taint, they want to influence the immoral behaviour. Bankers are stigmatized for their extremely high bonuses or irresponsible profit seeking. Moral stigma targets the profession’s responsibility and assumes agency. That means a banker can, and should do things differently according to public opinion. Additionally, when greedy bankers start normalizing what they do, public opinion stigmatizes them even more, to make clear their behaviour is still not acceptable. This is illustrated by the Ralph Hamers case in the Netherlands. In March 2018, ING Bank proposed a salary increase of 50% for its CEO, but the bank had to reverse the decision due to public disapproval. Newspapers had headers like: “One million extra? We do not accept.” (NRC, 14 March 2018). It was considered very inappropriate behaviour, thus adding to the moral stigma the bank carried already for its role during the financial crisis, and ING Bank lost many clients that month.

Whereas normalization seems helpful when work is dirty due to physical hardship and toxic elements as experienced by miners and firefighters, normalization seems less effective for morally tainted managers due to their assumed agency and responsibility. As a consequence, managers might feel inclined to cope with moral taint differently than only by normalization. While normalization might serve individuals in the short run by reducing their own experience of stress, society could see normalization as a variant of moral disengagement [27], thus adding fuel to the fire, and reinforcing the stigma of morally dirty leadership.

To explore the puzzle around the appropriateness of normalization as response to moral taint, we drew on moral leadership literature, which has studied the dynamics between moral leadership and reputation. Scholars like Rhode [28], Schminke et al. [29] and Zhu et al. [30] have explored how moral leadership can prevent a bad reputation. However, despite the fact that dirty work and moral leadership literature both study responses to moral taint, these responses have not been related (cf. [24,26,28,30]). As the effects of normalization can be counterproductive in situations of tainted leadership, we expected to find moral leadership responses in such cases as alternative response to normalization. However, it assumes that managers have sufficient agency to be able to make a difference in their institutional context, and that they intentionally try to prevent the creation of moral stigma. Following up on these assumptions, we explored how consultant managers cope with the morally dirty aspects of their overly demanding leadership style by studying both their normalization and moral leadership responses. To answer our question, we performed interviews with 12 consultant managers and with each one of their juniors about their common leadership experiences.

The study makes two contributions. First, we found that consultant managers illustrate several moral leadership tactics in their work, in addition to normalization. When talking about the existing social constructions of morally dirty leadership, they stress their moral leadership behaviour. This adds a new coping repertoire to the current dirty work literature (cf. [12,24,26,31,32,33,34]). Our research design does not allow conclusions about how effective this new coping repertoire might be in reducing moral stigma, or the stress caused by such stigma. Still, moral leadership is theoretically a more adequate response than normalizing as it does not imply moral disengagement, while normalization often does. As moral taint assumes agency, and responsibility for violating accepted moral standards, moral leadership is the response actually expected by society. When consultant managers meet this expectation better, it could reduce their feelings of stress together with the contempt in society. However, the agency of managers, and even more juniors, is limited, so both do still benefit from self-protection by normalization, and we found such responses as well.

Second, we have identified organization-level support for the moral leadership attempts of consultant managers. Currently, moral leadership literature heavily focuses on what a manager can do as an individual [28,30], but this ignores the limited agency of consultant managers. They need to respond to deadlines, client demands, top management expectations and other institutional constraints. The organization can offer support to counterbalance such constraints. Both junior consultants and their managers mention high-frequency performance reviews to monitor juniors, standard training and coaching sessions for juniors and policies to better select candidates for the job. The latter policies aim at what Ashforth et al. [35] call congruence work. We also found acknowledgement and compensation policies, such as ad-hoc time compensation, increased time off after periods of intense work, and flowers or other reward symbols, to say “thank you” after extraordinary performances. The institutionalized character of these support measures make them quite visible, which responds to the stressful image of consulting work and its pressuring management. The support measures imply visible acknowledgement that the work context challenges consultant leadership more than direct managers can handle on their own with individualized arrangements [18]. A similar multi-level management approach to improving employee wellbeing and to reducing stress has been developed in Australian universities [36].

### 1.1. Morally Tainted Work

Occupations are regarded as “dirty” in society when they defy accepted societal norms and values and therefore become stigmatized [26] (p. 414). The dirtiness can be physical, leading in extreme cases to disgust and repugnance, but it can also be social and moral [1], leading to a less physical form of social disapproval, but still loss of dignity. The dirtiness becomes more a metaphor then. In that sense, we disapprove the work of morally tainted occupations like used-car salespeople, tabloid reporters, exotic dancers, sex-shop workers or correctional officers (see [24,26,31,32,33,34]). It also does not imply everyone avoids these services, as some might even like them. High-status professions can be morally tainted as well: for instance, lawyers [14,15], healthcare professionals doing abortion work [37] and after the 2008 financial crisis, we can add bankers to the list [2,3]. 

Some scholars argue that moral stigma gives the “dirtiest” taint [31] (p. 100), [33] (p. 32). That is because physically or socially tainted work is usually protected by a necessity shield: garbage needs to be collected although it is dirty [38], we really need AIDS workers even though many could feel uncomfortable in their work context [39], and we also need firefighters although the work is dangerous [33]. In contrast, society sees more evil than necessity in morally dirty work. Morally stigmatized occupations can therefore experience high levels of entitativity, inducing a division of “them” versus “us” [40], (p. 626), which poses a strong “identity threat” [31], (p. 86).

In most dirty work studies, society is assumed to stigmatize a profession as with one voice. However, specific interest groups may be most active in socially constructing a stigma. For instance, these who like to smoke and are still healthy will most likely not actively co-construct the tobacco industry as morally tainted, but the anti-tobacco lobby will certainly do. Next to different interests, time has its effects. For instance, public opinion turns more and more to the acceptance of abortion work in spite of the downsides, thus softening the stigma, whereas the stigma around bankers shows opposite dynamics. While dirtiness of an occupation is reflected in the public eye, we still need to ask who really cares. For instance, the paying client of consultants does not seem to be very concerned about their more dubious virtues, as consultant services continue to be in demand. In contrast, client employees do identify with the popular criticism that consultants lack expertise [5]. Therefore, compared to the Western societies of the fifties and sixties where Hughes [1,23] and Goffman [22] published their seminal work, in our more diverse societies, some more nuance seems required in identifying which groups construct work as dirty and how widespread a stigma becomes.

### 1.2. Normalizing Morally Tainted Work

Feeling stigmatized usually leads to stress, and thus loss of “coping resources” [41] (p. 572). Hobfoll found that people can respond to such stress by trying “to retain, protect, and build” [42] (p. 516) their coping resources such as a positive sense of themselves, self-esteem, and socioeconomic status. Ashforth et al. [24] have found several tactics that workers and their managers utilize to protect their self-esteem, by normalizing a dirty work experience. They characterize the tactics as occupational ideologies, social buffers, confronting clients or public, and defensive tactics. In later work, Ashforth and Kreiner [31] assume some variation in the applicability of these tactics in relation to physically, socially and morally tainted work. Not all tactics appear equally useful to normalize moral taint.

Occupational ideologies help reframe, recalibrate or refocus the meaning attached to a dirty profession [26]. Reframing heightens the positive side(s) of an occupation. Recalibration revaluates the standards used to assess the “dirtiness” of the work by emphasizing that standards have changed. Refocusing shifts attention from tainted aspects of a profession to non-tainted ones. Occupational ideologies apply very well to moral taint, as Vaast and Levina [3] (p. 84) found in their study on retail bankers, Tyler [34] (p. 1490) identified in her study on sex shop workers and Tracy and Scott [33] (p. 26) revealed in their study on correctional officers. 

Social buffers help to gain validation from people who affirm the social worth of the tainted profession. However, for a morally stigmatized group, it might be hard to gain social support from people outside the group; therefore, the tendency will be to turn to in-group members. Ashforth and Kreiner [31] (p. 92) expect this to happen most often in cases of moral taint, but the tactic is not reported in the study by Vaast and Levina [3], maybe due to the high status of bankers. Tyler [34] (p. 1491) does find the tactic in her study on sex shop workers.

Confronting critical clients or the general public occurs when dirty workers actively indicate society’s perceptions of the occupation are wrong, by referring to opposite facts. Other methods to mitigate taint include “confrontational humour” and “counter-stereotypical behaviour” [24] (p. 162). However, Ashforth and Kreiner [31] do not mention confrontation tactics as effective in relation to moral taint, and also Vaast and Levina [3] explicitly wrote they do not find them. Thus, confronting outsiders seems less effective for normalizing moral taint, but the reason still remains an open question.

Finally, defence is a normalization tactic that appears very suitable in case of moral taint. Ashforth et al. [24] distinguish seven methods. The first is *avoidance* or the refusal to mention or observe dirty aspects of stigmatized work. The second is *gallows humour*, which is used to relieve the stress caused by the taint itself. The third is *accepting* and involves lowering one’s expectations. The fourth is *social comparison* in which the tainted profession is compared to jobs or previous times that are or were even worse. The fifth, *condemning condemners*, is a reversal of the criticism towards those who are judging the dirty workers. The sixth is *blaming and/or distancing from clients*, who are criticized for being the cause of the taint. The last, *distancing from role*, occurs when stigmatized workers separate their personal identity from their work identity. Ashforth and Kreiner [31] (pp. 93–100) suppose that condemning condemners and organization-level defences are most effective in case of moral taint. In contrast, Vaast and Levina [3] (p. 84) find that retail bankers heavily utilize the tactic of social comparison, and they find some new defensive tactics as well: *passing the blame on to other groups* (most often found tactic), *circumstantiating* (there are many reasons that can explain what happened), *diverting conversations*, and *conceding negative changes in the occupation* (thus claiming the essentials remain untainted). The list of tactics suitable for normalizing moral taint might be even longer, as research in this field is still nascent.

It is specific for moral dirtiness that society assumes responsibility and agency for harming accepted values and principles. Moreover, the more agency a worker or manager has, the dirtier moral taint becomes. For instance, Roca [43] (p. 139) argues, “the chief executive officer (CEO) of a tobacco company, who gains riches by endangering others’ health, might be perceived even more negatively than a blue-collar worker employed by the same company.” This aspect of agency in triggering the construction of moral taint is currently underexplored, as the focus has been more on how those who feel the stigma—the victims—can protect themselves against the stigmatizing outsiders [24]. Additionally, in the case of banking, agency and occupational stigma are clearly linked and the same applies to used-car salespeople consciously concealing flaws of the cars they sell. We expect to find this agency also for over-demanding managers in stressful service industries such as consulting or law firms [13,14,15,16,17,20,44]. When moral taint is socially constructed, it is a response to intentional behaviours: bankers who cause the financial crisis, and continue to demand their bonuses, or managers in law and consultancy firms who earn more money by consciously pressuring their employees beyond their limits. We wonder why literature on dirty work has not explored this agency, and the possibilities suggested by moral leadership theory to do things differently, and maybe even to prevent or moderate the taint.

### 1.3. Can Moral Leadership Moderate a Dirty Leadership Reputation?

While normalization focuses on how stress due to perceptions of dirty work can be reduced, not much attention has been given to acting on moral stigma as a social construction (cf. [3,24,33,34]). However, in some work, this seems quite well possible, and if it concerns moral taint, it is even demanded by society: the bonus culture in the banking industry is no necessity, and in consulting and law firms, management has sufficient agency to decrease the pressures they impose on their workers. There is not the kind of necessity as with physically tainted jobs like firefighting or cleaning [31]. Further, even in these jobs, we have acted on the dirtiness with technology, which has made several blue-collar jobs less dirty over time. For instance, technological innovations improving protective suits have reduced health risks for firefighters, or for those cleaning up asbestos. As health risks reduce, the reasons for socially constructing dirtiness lose impact as well.

Still, do middle managers also have these possibilities in case of dirty leadership, when performance standards are high, and clients and top management are very demanding? Howard discusses some options, starting from the assumption that leadership is a “process of communication, verbally and non-verbally, which involves coaching, motivating/inspiring, directing/guiding and supporting/counselling others” [45] (p. 385). Following up on similar studies as for instance by Stone et al. who argue that “the most effective leaders pay most attention to employees” [46] (p. 356), moral leadership aims at giving such attention [30].

The first option discussed in moral leadership literature is setting a moral example [29,30,47]. Aronson [48] (p. 245), for instance, argues that if leaders set “moral examples”, it fosters high levels of true motivation and morality overall, as employees look for an example they can follow. Applied to a high-performance setting, a lower-level manager could show his employees how to say no to a higher-level manager when managing own work pressures. However, in itself, a pressuring middle manager does not easily qualify for being a moral example. A second option often discussed is giving support, compassion and actively caring about others [29,30,49,50,51]. Treating employees with dignity and humanity will likely have positive effects on their performance [28]. Especially when management puts high pressures on employees, an open eye for their wellbeing and a supportive attitude might help not to overburden them. A third option of moral leadership is increasing your approachability [29,49]. That is considered to be crucial for establishing an “open environment” [50] (p. 164). When pressures are high, approachability in an open environment invites employees to speak up when they feel they reach their limits. Approachable, forgiving management leads to more employee wellbeing, more trust, and more sharing of interests between managers and their employees [49,52]. Fourth, in an open work environment, employees are more likely to engage in social control [49,50,51,52]. This results in more positive relationships among co-workers [29]. Co-workers could then feel more responsible to signal that colleagues get overstretched. Finally, when employees get the responsibility of performing tasks independently, psychological empowerment takes place [28,48]. Employees are being intrinsically stimulated to perform well and feel less commanded [30]. However, this is a risk as well, as you can easily push yourself too far in a high-performance culture, out of commitment. The five approaches are discussed as mutually supportive. For instance, if employees do not feel support from their manager, it is difficult for them to bring up their issues and to take responsibility [28,49].

Moral leadership approaches can potentially help to prevent or moderate a dirty work reputation originating from over demanding management. That is important, as high stress levels over extended periods can lead to emotional instability and decreased wellbeing at work [53] (p. 338). If then management is not approachable and does not foster an open culture, burnout and other stress-related diseases follow more easily, especially in the context of knowledge-intensive industries [54] (p. 166). That moment the dirty work image gets reconfirmed as well, with managers carrying the moral stigma. However, if and how middle managers in a high-performance context can execute moral leadership has not been researched yet. It is an open question if for instance consultant managers have sufficient agency to influence their morally dirty image this way, as they themselves are under high pressures as well. However, if some of these moral leadership approaches would work in their high-performance context, it might offer a more sustainable and more effective solution to the problem of their tainted leadership than only normalizing for themselves a situation society still considers dirty. If the agency of lower-level managers falls short, normalization is still the most likely thing left to do. Therefore, we expect a combination of normalization and moral leadership tactics when middle managers try to cope with the dirtiness of their leadership.

Based on our review of the literature, our main proposition is that the more agency a worker or manager has, the more likely it is that moral leadership tactics will be added to normalization tactics in order to cope with moral taint. Normalization is only a short-term solution for the worker and the morally tainted manager, whereas moral leadership can offer more fundamental answers to the problems that create a morally tainted leadership image.

## 2. Materials and Methods

### 2.1. Research Context

We chose management consulting as our research setting because it is well known that consulting managers put a lot of pressure on their employees. Alvesson and Robertson observed, for instance, that consultants frequently work more than 60 h a week [55] (see p. 221). Additionally, Gill found that promotions can only occur through high commitment, so workers constantly feel anxious about their current status and performance [44] (see p. 309). It makes consulting an extremely demanding profession with high levels of stress and burnout [17,20,56]. Society views such high demands and their negative health effects on consultants as “defying morality” [57] (p. 807). In fact, anonymous critiques indicating morally dirty leadership in consulting abound on public Internet forums (see a summary of these critiques in Table 1).

Thus, in the public eye, consulting managers are seen as very demanding in several ways. Such socially constructed dirtiness is also visible in the television series House of Lies, loosely based on a novel by Kihn [58], and we see leadership in consulting criticized in autobiographical accounts of ex-consultants as well [59,60]. These worries are confirmed in several academic studies on consultants’ work life [16,17,19,21,44,55].

### 2.2. Research Design and Sample

In order to explore moral taint assigned to consulting managers, we performed 24 semi-structured interviews. The interviews were conducted with 12 consultant managers, sometimes also called senior consultants, and one associated junior consultant each. The dyadic design helped to compare interpretations between juniors and managers on leadership experiences and its dirty nature. More than half of the interviewed consultants work at big international firms, the others at consulting firms mainly working for the Dutch market (small- and medium-sized). Specializations are diverse, as indicated in Table 2.

We selected respondents through “convenience and snowball sampling” [61] (p. 127), with the first few interviewees recommending possible candidates at other firms, mostly starting with the juniors and then connecting to their managers. Juniors and managers with the same number work together.

### 2.3. Interview Procedure

The interviews lasted an average of 45 min, ranging from 30 to 60 min. We offered anonymity, requested permission to record and started with a short personal introduction of interviewers and interviewees. All interviewees were informed about the study beforehand and gave their informed consent for inclusion before they participated in the study. The study was conducted in accordance with the guidelines of the School of Business and Economics at Vrije Universiteit Amsterdam. We explained to the interviewees that we would talk about tensions in the manager–employee relationship, based on three jokes (two of them were cartoons, and one was a text joke). The aim was to explore how consultant managers and their juniors experience the dirtiness of the management pressures. The three jokes were a starting point for doing very open interviews, in which we discussed each joke (see Table 3) for 5–15 min. The first one resonated most with the experiences of consultants resulting in long conversations, with the last one being the least.

As jokes do not present the truth literally, it helped us to introduce our topic in a stimulating, but when reflecting on it, not a leading, but rather a very open way [62]. Interviewees were first asked to interpret the jokes (for instance junior 9 said, “cartoon 1 is exaggerated.”), then they could explain if or how the jokes related to their work contexts (“this (80 h a week) rarely happens here”), and what further associations they had. Most respondents recognized aspects of the dirty management style illustrated in cartoon 1. Related to cartoon 2, the first response of junior 1 was: “What do they mean by this? That you always need to be happy at work? Or that you should pretend you are happy? With that I agree, as you don’t want to show your boss you don’t feel happy.” Many respondents recognized aspects of cartoon 2 due to their own personality or an “up or out” culture in their consultancy. For the same reasons of personality and company culture, others felt less connection to this cartoon. The third joke was hardly representing how respondents felt about what they have to do, and they did not recognize this dirty image of the job, like manager 2, who said, “I would put banker here instead of consultant.” All consultants stated they were proud enough to tell what they do, and did not recognize the suggested shame for being a consultant. However, some did refer to other “sick stereotypes” they encountered, like junior 9 who went on holiday, introduced herself as a consultant, getting the question in return, and said, “where is your lease car and credit card?” The quoted interpretations illustrate that respondents made sense of all three jokes in their own way, by referring to their own experiences.

After this free interpretation, the interviewer facilitated a broad discussion including probing questions concerning over-demanding managers, observed critical evaluations of the behaviour of consultant managers, the experienced effects of their leadership style, and how managers and juniors where coping with the situation of pressuring leadership and its morally tainted nature. None of such coping was suggested in the jokes, with only the pressures and the reputation proposed. Starting a conversation with a respondent by asking for interpreting three jokes is new, but doing open explorative interviews aligns with prior research on dirty work (cf. [3,24,33,34]). It is a good way to explore experiences with work pressures, leadership and dirtiness, and it fits our nascent field of research as outlined by Edmondson and McManus [63] (p. 1170).

### 2.4. Data Analysis

To analyse the interviews, we worked mostly abductive. We applied elements of a grounded theory approach in our coding [64] but also used existing dirty work and moral leadership tactics to interpret the data. We kept an eye out for any codes that did not fit the existing theoretical labels. To do so, the transcribed interviews were coded with the qualitative data analysis tool Atlas.ti (ATLAS.ti Scientific Software Development GmbH, Berlin, Germany). This resulted in 814 relevant codes with data-driven summarizing labels mostly connected to one quote only, and incidentally to two. Both authors coded iteratively and pointed upon which they disagreed with were discussed and then aligned. An overview of all codes can be found below in Table 4. In the results section we present codes related to dirty leadership pressures and related effects in Table 5, codes related to normalization tactics in Table 6 and codes related to moral leadership tactics in Table 7.

**Figure 1:**
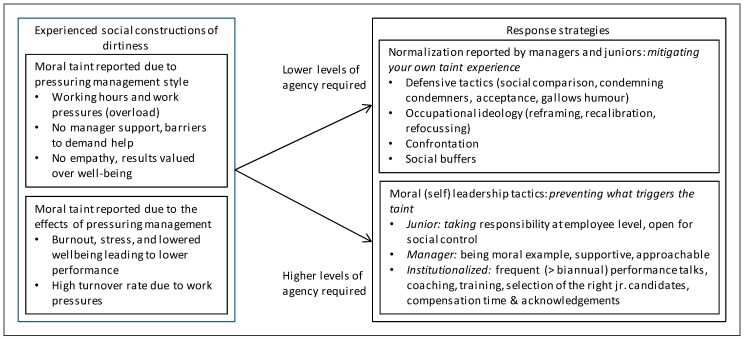
Moral taint constructions linked to management, and response strategies.

The leadership pressures and their effects on juniors were coded as morally dirty based on two ethical perspectives: deontology (pressures) and consequentialism (effects). Criticized stressors, such as long working hours and high work pressure, were coded as morally dirty from a deontological point of view. For example, demanding more hours than allowed by law does not conform to duty [65]. Codes identifying criticized negative effects, such as burnout, decreased wellbeing, or high turnover rates caused by health problems, were coded as immoral from a consequentialist perspective [66]. Normalization and moral leadership tactics were labelled with existing concepts from the discussed literature, except for the new ones that emerged from the data. Our findings suggest that moral taint experienced by consultant managers is not only mitigated by taint normalization, but also by known and lesser known moral leadership tactics. A process model summarizing the main codes and sub codes (in the boxes) and their relationships (arrows) is presented at the start of the results section (see Figure 1).

## 3. Results

Figure 1 presents a conceptual model representing our coded categories. The model illustrates how pressures due to leadership are experienced as morally dirty by both junior consultants and their managers, and how this experience of moral taint invites on the one hand normalization responses as predicted by dirty work literature, but on the other hand also moral leadership initiatives to prevent or moderate the negative effects of leadership pressures. The reported forms of moral leadership we found require different levels of agency, and are relate to juniors, managing consultants and those who can design institutions at consultancies. We first reported leadership pressures experienced as dirty, second normalization tactics as coping response and third moral leadership practices as an alternative response, as seen from the perspective of managers and junior consultants.

### 3.1. Moral Taint Due to Pressuring Management

The interviews revealed stressors and effects due to pressuring management that interviewees perceived as morally tainted. Respondents expressed their interpretations of moral taint quite explicitly through negative judgments or more implicitly: facts were given and the audience was left to pass judgment. Table 5 summarizes the shared interpretations of managers and junior consultants, and gives for each of the codes their groundedness (how many quotes we could label with the same code) and an illustrative quote of both managers and juniors. It is important to note that the pressuring management style is criticized substantially more often than its negative effects.

Statements from both junior 7 and manager 12 in Table 5 indicate it is quite common in consulting to be asked to work up to 60 h a week and incidentally up to 80 h a week. This is substantially longer than the Dutch maximum of 40 h a week. For a period no longer than 16 weeks, Dutch labour law allows workers to work up till 48 h a week on average [67], but consultants are asked to work much longer. Because of projects with overlapping deadlines, pressuring managers and demanding clients, junior 11 (Table 5) compares his work environment to that of a “pressure cooker”, suggesting the pressures are far from comfortable. Of all dirty leadership pressures, required work hours are criticized most by the juniors, and managers admit the pressures are as high as the juniors indicate. It makes the management style morally tainted; for instance, junior 10 said (laughs while looking at cartoon 1), “This is anonymous? Yes, this applies to my manager! This is quite bad indeed. But I need to add some nuance. I recognize this, but it is also something I want to do. I chose to work the 60, 70, 80 h. And I seek challenges, new clients, personal development, etc. This works bi-directional.”

While the pressures mentioned above can also be attributed to the work context, and not only to the manager, juniors specifically mention the formal distance they can feel between themselves and their demanding managers. When facing difficulties, juniors can feel “ashamed” for opening up, sensing it is better not to “lose face” by admitting they struggle with the work pressures (see junior 4 in Table 4). Managers recognize the experience of this distance (like manager 3 in Table 4) and admit “you often discover it (overload struggles) later than their direct environment”.

Related to this is the focus on results. Consulting firms are organized around meeting productivity and sales targets, causing managers to be primarily concerned with the productivity aspect of their juniors’ performances, and the cost of their juniors’ wellbeing. As a result, juniors criticize the aspect of being treated as a source of profit. Junior 10 (Table 5) explains it is key that the “client is happy”, and feels that it is a “dangerous criterion”, as it can push you too far. Managers confirm this, and admit that “consulting is a hard environment” (manager 9), which adds to the list of morally dirty aspects in the leadership style.

The juniors and managers not only criticize the moral dirtiness of the cold management style with the focus on results and low tolerance for personal failure. To a lesser extent, they also criticize the immoral effects of such high pressures. Burnout is mentioned most often, and also qualified as the most negative consequence. Manager 3 (Table 5) indicates that increasingly young colleagues suffer from burnout. In addition, junior 10 admitted that she had suffered from a burnout herself. Although we cannot quantify based on our interview data, literature on consultants does indicate a high prevalence of burnout, stress and related psychological problems among consultants, in line with our findings [17,19,20,56].

Some workers, rather than having a burnout, share that they become mentally imbalanced, feel depressed, or have negative emotions. The quote from manager 5 in Table 5 illustrates how stress reduced his performance and work satisfaction. At times that too many stressors escalated “you couldn’t care less about performance”, a finding also observed by Espeland [68] (see p. 180).

A related effect criticized by our interviewees is the high turnover rate among juniors. It is seen as response to the extreme demands they face. The quotes from manager 12 and junior 11 in Table 5 indicate this, as you “cannot let juniors work that many hours” (manager 12). “They will ask if this is the right job for them, and then they leave.” (junior 11). These critiques again indicate dirty leadership, and question the sustainability of the work for juniors.

Juniors and consultant managers are surprisingly aligned in their judgements of when and where their occupation crosses the borders of acceptable work demands. They clearly articulate which leadership pressures and effects are unacceptable against the norms and laws in society. These internalized critical social judgements give stress, as discussed in the dirty work literature, in addition to the work pressures themselves. Therefore, the motivation to normalize an experience of moral taint will be higher, the more conscious you are about the critical public and peer judgements. Both the juniors might normalize (they do not like to be seen as a victim) as well as the pressuring managers (who do not like to be seen as over-demanding).

### 3.2. Normalizing Morally Tainted Management

If members of an occupation feel aspects of their work are perceived as morally dirty, they are found to engage in normalization to protect their self-image [24,31]. The occurrence of normalization signals foremost a perception of taint. By using normalization tactics, the interviewees tried to mitigate their own experience of being seen as morally tainted, as this causes stress. Our interviewees applied several normalization tactics when discussing their leadership experiences. Table 6 shows that the normalization tactics are well grounded. Remarkably, juniors illustrate normalization more than managers.

Instances of taint normalization illustrated in the interviews were most often defensive, with social comparison applied the most. Other forms of defence included condemning condemners, acceptance, and a few instances of gallows humour. Manager 2 in Table 6 demonstrated the use of social comparison by relating the moral reputation of consultant managers to, in his eyes, the worse reputation of some other professions: “Bankers have a bigger problem.… Lawyers as well.” Condemning condemners was used to normalize consultants’ long working hours. Compared to her own schedule junior 10 considered working from 9 to 5 “more of a regime”. This defensive normalization intends to mitigate the feeling of moral stigma due to leadership pressures put on you: with long working hours, you can still feel better off than the 9-to-5 employee.

Confronting public opinion was a second normalization tactic repeatedly used by juniors and managers. With this tactic, someone proactively confronts the public’s perception of occupational taint, intending to change the view. Junior 1 and manager 5 in Table 6 tried this by correcting the stereotypical belief that consultants always work 80 h a week, as suggested in one of the cartoons. Junior 1 argued this is exaggerated and manager 5 stated that it is impossible: “Look, I work from 8 a.m. till 7 p.m. That is 55 h. To make it 80 h would mean I could not sleep anymore.” We thus find opposite opinions: many consultants complain about workweeks up till 80 h, as illustrated in Table 4, while manager 5 denies it even as a possibility. Still, he does admit a 55-h average workweek (not counting the weekend)! Another theme for confronting public opinion is the lack of humanity in consultant leadership due to the results-oriented work culture (see Table 5). Consultants are confronting the universality of this tainted aspect of their work suggested in the second cartoon, but less so than the 80-h figure from the first cartoon.

Third, occupational ideology tactics were practiced. Consultants transformed negative opinions about their profession into more positive ones, by reframing, recalibrating and refocusing. Junior 6 recalibrated long working hours and high workload when he stated people “do great work because of that”; this recalibrated the extra effort as simply needed to reach the intended effects. Furthermore, manager 4 reframed the harsh conditions juniors face by emphasizing that they are “helping others”, thus presenting the efforts of juniors in a different light. Examples of refocusing included shifting attention to aspects of the work that made consultants proud or happy, like their impact, their pay or their status.

Although creating social buffers supports in-group protection, we found it rarely used to normalize the high-pressure work context. Manager 3 and junior 8 made a distinction between “us” versus “them” as outsiders: “us, young professionals, we appreciate it”, or “my wife and I, both consultants, we understand” (see Table 6).

While we interviewed an equal number of managers and junior consultants, the juniors illustrate normalization tactics more. As juniors could be seen as victims with a low degree of agency, normalizing can help. They need to give their best efforts in order to survive. Still, they have a responsibility for their own health, actually more so than their managers. They also have the agency to choose for the job, and they can quit. Managers have more influence: although they have to play their part in the up or out performance system, they are also the ones pressuring their juniors. Managers can make a difference, but their agency to prevent an output oriented and pressuring form of leadership has limits as well, which makes normalization still a convenient way out.

### 3.3. Moral Leadership to Prevent Moral Taint

Whereas normalization mitigates the experience of stress caused by the feeling that you have a dirty job, interviewees also tried to prevent a morally dirty image by influencing the effects of high work pressures and the extreme focus on results. We have coded many of these prevention tactics as moral leadership because they exactly match the tactics mentioned in this literature. However, institutionalized forms of support for juniors, like frequent performance talks, acknowledgement policies and tailored selection procedures (italicized in Table 7), were new to moral leadership literature. Still, they fit the same rationale of preventing the criticized consequences like burnout or emotional imbalance, and of counteracting the impression of immoral values in the leadership style like the strong output orientation and lack of humanity. Additionally, they help managers to execute traditional moral leadership tactics by organizing how and when they give attention to juniors.

#### 3.3.1. Traditional Moral Leadership Approaches

Managers most often mention the importance of compassion and support for their juniors, which aligns with moral leadership theory [29,30,49,50,51]. It implies managers not only try to be actively aware of the stressors they put on juniors, but also make them discussable. In our interviews, managers emphasized the importance of actively approaching juniors, especially those who seem stressed. Manager 8 claimed she “always asks them a lot of questions”. Her junior (junior 8 in Table 7) confirmed this.

A second aspect, also mentioned in moral leadership literature, is an ‘open culture for social control’, as it creates positive social effects [29] (p. 139). This is relevant, as consultants mostly work together in project teams [69] (p. 559). Our interviewees illustrated how managers help to establish an open culture, in which peers are encouraged to express their feelings to each other. Manager 10 (Table 7) stated how important it is to create an environment in which everybody can ‘speak up’. Her junior (10) confirmed the open culture and the social control: “I experience the social control. It means there is sufficient attention for the persons themselves, and how they really feel, instead of only a result focus, this extreme focus.” Junior 9 (see Table 7) has a similar experience of social control, and mentions that colleagues take care of each other.

Third, managers try to be approachable and to react with understanding and forgiveness if juniors approach them. Manager 10 stated that if a junior dared to approach her, she would definitely listen and then try to manage the problem. Manager 4 illustrated a similar attitude: “You try to find out what is the matter, and then seek for a solution together. That cartoon suggesting to dismiss them immediately is not our approach, and I would not support it.” His junior confirmed the approachable “the doors are always open” and the forgiving approach of his manager (junior 4, Table 7), but he knows other stories as well, where you “first need to book your appointment”. 

The impact of acting as a moral example is sufficiently discussed in the literature, as documented in our theory section. However, the tactic was hardly mentioned by our interviewees, indicating it is a difficult one in the context of consulting. Manager 11 (Table 7, bottom row) acknowledged that juniors find it difficult to open up about stressors, and yet he expressed that if he became open about himself, “it is easier for people to open up also”. Only one junior (junior 9) referred to exemplary behaviour of one manager who did not respond to an email she had sent on a Sunday. That manager explained later that the weekend should be weekend. However, this does not seem to be standard practice in this occupation.

#### 3.3.2. Juniors’ Role in Making Moral Leadership Work

The fourth moral leadership tactic we found (based on groundedness) was giving responsibility to the employee, a practice most often expressed by juniors. By making the juniors more responsible for how they perform their tasks, their feelings of helplessness and lack of control can decrease [30]. Manager 4 (Table 7) stated that he tries to foster autonomy of juniors by not getting involved in their daily tasks. Giving autonomy to plan his or her own schedule and projects reduces the negative impact of workload and deadline stress. Juniors are supported to develop this autonomy, as mentioned by junior 6, and such autonomy is indeed expected. Manager 6 explains: “If they have a problem, they should call me. Sometimes at the end of a call, they just thank me for the talk. It can help to better make sense of a difficult situation. But they have to approach me, as we discussed before. And that does not always happen.”

#### 3.3.3. Institutional Approaches to Moral Leadership

Respondents also referred to institutionalized practices, such as the monitoring of juniors through monthly or quarterly performance reviews next to the annual talk. Junior 2 mentions that her consultancy organizes different types of formal evaluations every year (see Table 7), and elsewhere in the interview she refers to evaluations “after every project”. It gives juniors a platform to speak up, and this way it is institutionalized that they receive sufficient attention from their managers. Trainings are institutionalized as well, as illustrated in the quotes from juniors 2 and 9 in Table 7. Examples are work–life balance workshops and personal development courses that better prepare juniors to handle the work stress.

Additionally, consultancies make use of an extensive selection process, aimed at hiring these juniors who are sturdy enough to handle the stress of being a consultant, as explained by manager 9 in Table 7. Some people like a challenging work environment and are able to handle the lack of structure for many years. However, the work is too demanding for many others, so consultancies are aware of the importance to pick the right people, in order to protect their reputation as an employer and to manage the consequences of the severe work pressures up front.

A second group of institutionalized practices to counter the moral taint of pressuring management includes compensation and acknowledgment. Overwork or high pressure is not compensated by additional pay, because it is seen as part of the job. However, managers often give juniors visible recognition after a stressful period. For instance, juniors are given dinners, social events, flowers, a couple of days off and even vacations. The quote from manager 3 (Table 7) illustrated this practice. The manager acknowledges the stress, and marks it as out-of-the-ordinary. In addition to acknowledging the stress, he compensates his juniors with time to recover, ultimately also hoping to prevent severe consequences. His junior confirmed his work is quite intense now, heading towards the end of several projects, “but you also know you can slow down after the deadlines. That is quite accepted” (junior 3). Junior 6 illustrated another practice explained by manager 3: “we often hear: ‘thanks for your help, you did really well’.”

The combination of traditional, more personal, and consultancy-specific institutionalized practices to support juniors demonstrates that the direct managers are not the only ones who take responsibility for supporting juniors. The organization as a whole has taken action to prevent escalation of stress. These institutionalized practices aim at making the consequences of the stressors less severe and the management more humane, at the same time reducing perceptions of moral taint in the eyes of the juniors and their managers. Such multi-level moral leadership is potentially a more effective approach to coping with perceptions of morally tainted leadership than taint normalization. Normalization only targets at the stress due to perceptions of taint, which is symptom management. Moral leadership targets the specific causes behind these perceptions. Additionally, given the fact that the pressuring leadership style is far more criticized than its effects (see Table 5), the multi-level approach seems promising.

## 4. Discussion

In summary, we found that moral leadership approaches were discussed quite a lot when compared to normalization responses. Twice as many quotes (350 vs. 171) illustrated moral leadership tactics in response to the dirty leadership images. When reflecting on moral leadership approaches, juniors and managers emphasized different options. Managers mentioned their active compassion and support of juniors twice as often as juniors, whereas juniors mentioned the approachability of their managers more often as important to them. Still, juniors and managers referred to the same kind of tactics. This, together with the ample groundedness of the codes, indicates good saturation.

Table 5 shows that the leadership style of managers is more often constructed as dirty (246 quotes) than the resulting effects (37 quotes). However, the number of quotes is not very conclusive regarding this dirtiness as something “essential”. The mentioned effects like burnout are really problematic and not mentioning it might even be an avoidance or denial strategy. Still, there is a lot of talk about the dirtiness of the leadership style and this is a big issue in the construction of consultants’ dirty leadership.

Moral leadership aims at neutralizing such dirtiness. For instance, the impressions of an output-oriented and pressuring leadership style resulting in long working hours and high workloads is countered by traditional moral leadership tactics. They counter the stigma that managers do not provide support and have no empathy, as these traditional tactics are targeting exactly these aspects of dirtiness in the leadership style. Institutionalized moral leadership tactics, like regular performance talks, training, selective hiring and various compensation tactics, are also discussed a lot, which indicates their social visibility, with the construction of a better leadership impression. These institutionalized approaches seem specific for our high-performance setting, and might be relevant to other high-pressure contexts as well, such as investment banks or law firms. The applicability of the individual moral leadership tactics must be much wider, as these tactics have been found in many other work contexts already [29,30,49,50].

The findings of our study contribute to the literature on moral taint and moral leadership. First, we found that moral problems that cause a dirty leadership image can be targeted with moral leadership approaches [29,30,49]. Consulting managers engage in many forms of moral leadership to counterbalance what juniors and managers socially construct as a dirty leadership style. Especially the pressuring leadership style, like focusing on output only and lack of personal attention, is experienced as dirty in the image of consultant management. Traditional moral leadership tactics target such dirty aspects in the leadership style directly. Thus, they socially construct alternative and more ambiguous leadership image: pressuring yes, but also committed. As deeds can speak louder than words, this approach seems quite relevant for influencing a dirty leadership image. Except for confronting public opinion, normalizing mainly has a focus on individual stress reduction due to a dirty image. Our findings add a new repertoire of tactics to the literature on moral taint (cf. [3,24,26,35]). As a consequence, normalization tactics are just one way to respond to the experience of moral taint.

On a more critical note, we found that some of the propositions in Ashforth and Kreiner [31] did not hold very well in the context of consulting. For instance, proposition 4 that morally tainted professions are assumed to mostly engage in group-level defensive tactics, and proposition 10 that such professions create social buffers to normalize taint were not very prominent in our context. We found, similar to findings by Vaast and Levina [3], that the tactic of social buffering is not utilized much, whereas defensive tactics are most frequently applied. Considering defensive tactics Ashforth and Kreiner [31] assume in their proposition 11 that condemning condemners is the most common one to normalize moral taint, however, in our study, social comparison is the most reported normalizing tactic, a result that aligns by and large with the findings of Vaast and Levina [3] situated in the banking industry. Unlike the propositions in Ashforth and Kreiner [31], we found many instances of confronting public opinions about the dirtiness of consultant leadership. Probably, effectively normalizing moral taint largely depends on context, as both banking and consulting belong to the professional service sector. As professional service industries are a growing field of employment in today’s societies, future research should address these high-performance sectors more specifically when studying morally tainted leadership and the potentially tainted health consequences for employees, like burnout. It is important to study such consequences, and possible gender, role or seniority differences based on a quantitative research design, to better tease out to what extent normalization attempts cover up such consequences.

Second, we contribute to moral leadership literature by finding that consultant managers not only apply traditional moral leadership approaches like being approachable, compassionate supportive and encouraging. Consultancies also support managers with institutionalized measures that aim at moral leadership, and that are new in this literature (cf. [29,30,49,50]). As the agency of managers is constrained by institutional pressures, we see that the organization also creates counter pressures with several specific HR practices. As these HR practices are quite visible, they help to clean up the dirty leadership image, again with deeds more than words. Therefore, we invite moral leadership literature to better include the organizational and institutional levels in its theorizing. By having frequent performance reviews planned, trainings available, and several non-monetary compensation policies in place, consultant managers mentioned how they are supported in taking responsibility for the wellbeing of juniors. These institutionalized measures might be specific to high-performance occupations with demanding top management, clients and projects, where it is very tempting to satisfy client needs first and think about your juniors second [11]. Therefore, similar institutional support might be relevant to other high-pressure professional service contexts, such as investment banking, law firms and marketing agencies, or even at the more competitive top universities.

Our research has also some practical implications beyond the fields of moral leadership theory and dirty work literature. We consider it a promising and innovative combination to facilitate moral leadership of middle managers with supportive HR practices. Such tailored institutions might help protecting employee health and reduce psychosocial risks at work, especially in settings of knowledge-intensive work. These are high-performance work contexts where employees are often ambitious and willing to give their best, but where burnout risks lure around the corner, especially when employees do not feel they are seen and cared for, or rewarded for taking initiative and for acting responsible towards their organization, often at the cost of their own mental resources. Our research shows that HR institutions can be further developed to support middle management in taking care of their employees, inspired by moral leadership ideas. If organizations create such institutions to give attention, to show compassion and to take responsibility for workers that give their best, it could help to move away from the more bureaucratic, rule-based and one-size-fits-all HR institutions we are so familiar with today.

## 5. Conclusions

The identified moral leadership approaches on both the levels of individual manager and the organization add to our understanding of how organizations can influence their image of moral dirtiness associated with pressuring, output-oriented management. Similar multi-level approaches to support employees have been identified in Australian universities [36], and they seem promising in empowering middle management to become moral leaders. However, based on our research findings, we cannot answer yet to what extent individual and organizational moral leadership approaches in consulting make life of juniors really better (see [16,17,19,44]). It is possible that they are merely used instrumentally, to push performance of juniors just a little bit further, without irreversible consequences for leadership reputation and employee health and wellbeing. What we can conclude is that the morally dirty reputation of consultancies is not only articulated regarding the consultant–client relationships [7,8,9,10]. Moral leadership issues between consultants and their managers are also publicly addressed, and more prominently indeed than their moral leadership approaches.

## Figures and Tables

**Table 1:** Moral taint indications of over-demanding managers on Internet forums.

Critiques on Consulting Forums Found on Different Websites:	Threads/Entries	Period	Illustrative Quotes
Pressure of long working hours http://forum.top-consultant.com/ http://forums.whirlpool.net.au/ http://postgraduateforum.com/ http://www.wallstreetoasis.com/ (last assessed on 22 March 2017)	14/79	2006–2015	Forget work/life balance. Any big 4 [consultancy] you go to, **you’ll be overworked**. (User #41779, forum.whirlpool.net.au, entry 2007)
Heavy workload; deadlines http://forum.top-consultant.com/ http://forums.whirlpool.net.au/ (last assessed on 22 March 2017)	12/69	2004–2015	Your start-off salary will be excellent. **For the brain damage resulting over the years there**, they will not compensate. (User, forum.top-consultant.com, entry 2004)
Fear of boss; not supportive http://lynntaylorconsulting.com/ http://managementconsulted.com/ http://socialanxietysupport.com/ (last assessed on 22 March 2017)	11/50	2008–2015	I’m in a bad place at work. It’s in a high stakes consultancy firm, and my boss is a la Glen C. in Devil Wears Prada. Anyway, **my fear has just gotten worse**. (User, socialanxietysupport.com, entry 2009)
No empathy; focus on results http://forum.top-consultant.com/ http://lynntaylorconsulting.com/ (last assessed 22 March 2017)	8/24	2007–2013	The most heard story is about the boss who thinks that you can do anything in Excel with just a couple of clicks. **Never understands why everything takes so much time**. Also, never really knows what doing a job entails, and how all that analyst work on is done. (User, forum.top-consultant.com, entry 2007).

**Table 2:** Interviewee characteristics.

Consultant	Gender	Age	Years in Company	Own Hours per Week	Branch of Firm	Size of Firm *
Junior 1	Male	24	1	50–55	Marketing	Small
Manager 1	Male	46	9	45–50	Marketing	Small
Junior 2	Female	25	1.5	45–55	Healthcare	Medium
Manager 2	Male	46	15	45–50	Healthcare	Medium
Junior 3	Male	27	1.5	40–50	IT	Large
Manager 3	Male	42	13	70	IT	Large
Junior 4	Male	27	2.5	50–55	Corporate Finance	Medium
Manager 4	Male	35	11	50	Corporate Finance	Medium
Junior 5	Male	25	1	60	Strategy	Large
Manager 5	Male	30	5	50–70	Strategy	Large
Junior 6	Male	27	1.5	50–60	M&A	Large
Manager 6	Male	34	8	50–80	M&A	Large
Junior 7	Male	26	1.5	45–60	IT	Large
Manager 7	Male	30	6	40–80	IT	Large
Junior 8	Female	25	1.5	40–45	Strategy	Small
Manager 8	Female	28	4	45–60	Strategy	Small
Junior 9	Female	25	1	45–60	Human Resources	Medium
Manager 9	Female	35	8	40–60	Human Resources	Medium
Junior 10	Female	28	3	40–80	Innovation & Change	Medium
Manager 10	Female	35	6	40–80	Innovation & Change	Medium
Junior 11	Male	27	1	50–70	Strategy & Operations	Large
Manager 11	Male	37	9	50–60	Strategy & Operations	Large
Junior 12	Male	28	1.5	55	Strategy & Operations	Large
Manager 12	Male	48	4	50–60	Strategy & Operations	Large

* Number of employees in consulting departments based on http://www.vault.com/. Last accessed: 18 May 2016). Small: <100 employees; Medium: 100–500 employees; Large: >500 employees.

**Table 3:** Three consultant manager jokes indicating moral taint.

*Manager A in his office:* What are they complaining about…. The work is challenging, interesting, demanding! *Manager B:* AND we let them do it 80 h per week! Fran (2009) Retrieved from: https://www.cartoonstock.com/, accessed: 23 March 2017
*Manager A to Manager B when walking through the office:* Naturally our workers look happy. The penalty for not being happy is instant dismissal *Financial Times*, 20 May 2013. Retrieved from: https://www.ft.com/content/41f990f0-b955-11e2-bc57-00144feabdc0#axzz2U2zMvxmp, accessed: 23 March 2017
Please don’t tell my mother I’m a consultant. She thinks I play guitar in a strip joint. Consultant Jokes Retrieved from: http://www.weitzenegger.de/en/to/jokes.html, accessed: 23 March 2017.

**Table 4:** Parent, child and grandchild codes.

Parent Codes	Child Codes	Grandchild Codes
Morally dirty leadership	Dirty pressures	Long working hours and high workload No support; barriers to request help Focus on results instead of wellbeing
	Dirty effects	Burnout Decreased wellbeing & performance High turnover rate due to pressure
Normalization tactics	Defence	Social comparison Condemning condemners Acceptance Gallows humour
	Confronting	--
	Occupational ideology	Reframing Recalibrating Refocussing
	Social buffers	--
Moral leadership tactics	Individual tactics	Personal support by compassionate managers Open culture for social control Approachability of managers Responsibility given to employees Being a moral example
	Institutionalized tactics	Institutional support through selection of the right candidates, performance reviews & training Compensation time & acknowledgement policies

**Table 5:** Management-induced pressures perceived as morally tainted.

Category	Groundedness			Illustrative Quote
	Tot 283	Jr 153	M 130	
*Dirty pressures*	*246*			
Long working hours and high workload	111	63	48	“Yes, juniors work long hours. There are projects where they work for longer periods **about 60 h a week**.”—Manager 12. “Consulting is working from deadline to deadline. And if a deadline requires a lot, then **working 80 h occurs easily**.”—Junior 7. “Working here is working in a **pressure cooker**. It is just hard work. You have deadlines.”—Junior 11.
No support; barriers to request help	78	50	28	“**Often juniors are ashamed**, like, I am so young, why does it happen to me? As a manager you often discover it [overload struggles] later than their direct environment, and that it does not go well.”—Manager 3. “I know myself. I sure have my issues here. But **I would never go with those to my boss**.… opening up could be seen as a loss of face.”—Junior 4.
Focus on results instead of wellbeing	57	26	31	“**Consulting is a hard environment**. As a junior you have to satisfy your project managers. Failing to satisfy your manager can only happen 1 or 2 times. Then they look for someone else.”—Manager 9 “The key rule is: as long as the client is happy. And that can be a really **dangerous criterion**, in which you can easily go too far.”—Junior 10.
*Dirty effects*	*37*			
Burnout	18	5	13	“What I do see, is the age at which people come down with long-term illness is rapidly declining. I have an **increasing number of people under 30 coming to me with such symptoms**.”—Manager 3. “If you struggle with boundaries, and want to do everything perfectly, working as a consultant is not sustainable. And that’s what happened to me. **I made myself sick**.”—Junior 10.
Decreased wellbeing & performance	10	4	6	“If you are not handling them [the stressors of consulting] well, you see that in your performance. Then you **don’t even like working here, and you couldn’t care less about performance**.”—Manager 5. “If it is not your own choice to work 80 h a week. It is also not constructive, **for either you or your results**.”—Junior 10.
High turnover rate due to pressure	9	5	4	“In the moment you are like ‘Okay, we have to get through this’. But you know it’s not sustainable. **You can’t let juniors work that many hours for several weeks on projects**. You know that they will leave after a year or so. It’s not sustainable.”—Manager 12. “If people are really unhappy with their projects, they will ask if this is the right job for them, **and then they leave**.”—Junior 11

Tot = Total; Jr = Junior consultant; M = Manager.

**Table 6:** Taint normalizing tactics that mitigate the experience of moral taint in consulting.

Category	Groundedness			Illustrative Quote
	Tot 171	Jr 101	M 70	
Defence: mainly social comparison, also condemning condemners, etc.	64	40	24	“I think the reputation problem for consultants has become less over the years. **Bankers have a bigger problem**.… Lawyers as well, and medical specialists.…, why should the latter earn so much?”—Manager 2. “Yes, I don’t work from 9 to 5.… **These people have a mentality like, whatever**. That does not fit me. So, I don’t work from 9 to 5. But I would hate that.… Actually, I think that working 9 to 5 is more of a regime than working 80 h.”—Junior 10.
Confronting	51	23	28	“I made the calculations myself. Look, I work from 8 A.M. till 7 P.M. That is 55 h. To make it 80 h would mean I could not sleep anymore. **That is not how it works**.”—Manager 5. “I understand that cartoon saying we work 80 h, **but it is exaggerated**. Who is working 80 h…?”—Junior 1
Occupational ideology	46	31	15	“I really like consulting. What I like is to help others and explicate things. **The way I see consulting, is that it helps others**. So no way am I ashamed of that.”—Manager 4. “There are people here that can’t say no; they can’t stop. **But they really like that and do great work because of that**. They are actively seeking such pressure.”—Junior 6
Social buffers	10	7	3	“My wife and I, we both work as consultants, so **we understand each other** in terms of work and our careers.”—Manager 3. “When I told my uncle that I wanted to become a consultant, he.… was very negative. But I think, **among the young professionals, among us, consultancy is being highly appreciated**.”—Junior 8.

Tot = Total; Jr = Junior consultant; M = Manager.

**Table 7:** Moral leadership tactics used to prevent moral taint.

Category	Groundedness			Illustrative Quote
	Tot 350	Jr 181	M 168	
Personal support by compassionate managers	96	30	66	“I always **ask them a lot of questions**, like ‘What does your day look like? What are your responsibilities? What costs too much energy?’. With that, you intend to start something, and make the junior rethink himself.”—Manager 8. “I work around 60 h now.… They monitor that you do not work too much. … You have conversations like ‘you leave the project too late every time’. **That is your manager who talks to you individually**.”—Junior 8.
Open culture for social control	61	34	27	“It is very important to ensure that your employees dare to speak up, to create an environment in which people **feel safe**.”—Manager 10. “There are people that I see three times a week, who could assess my feelings better (than my manager). So I think it is the role of everyone: **social control**.”—Junior 9.
Approachability of managers	59	40	19	“I surely am **approachable**. And I am definitely open to those conversations (about stressors).”—Manager 10. “There is no barrier to approach my manager. If there were something bothering me, I could tell him. I also know other stories…. **Here the doors are always open**.”—Junior 4
Responsibility given to employees	58	42	16	“Everybody has their own responsibilities. Of course, I will have the final responsibility, but I don’t manage their daily activities…. **We give them free reign**.”—Manager 4. “In the beginning you get a lot of guidance.... Now, after 1.5 years, I am much more pro-active. I say I want to do this or that. **I organize and plan myself**.”—Junior 6
*Institutional support through selection of the right candidates, performance reviews & training*	48	25	22	“The other day, I conducted some job interviews, in which I explicitly asked: “What do you think of working over night?”.… **So I test them**, to see if they need structure or not.”—Manager 9. “We have an **HR (Human Resource) cycle**, in which we have a talk about performance, a talk on development and several training courses.”—Junior 2. “We recently got a case about work-life balance.… Here we got taught **how to say ‘no’** to managers.”—Junior 9.
*Compensation time & acknowledgement policies*	22	9	13	“‘If we require our employees to work on the weekends, we **compensate** for that. We send a gift coupon to the family, especially if it happens more often, or we send flowers. And if we require our employees to work hard for an extended period, we send them on a weekend trip with their family.”—Manager 3. “We often hear ‘**thanks for your help, you did really well**’…. After every project we go out for dinner…. Sometimes there also is an event and you get some award for your contribution (he shows an award), awards like that.”—Junior 6
Being a moral example	6	1	5	“People try to guard their image. But people should let that guard go. Saying ‘Okay, this is who I am; I am putting it on the table’. And then it’s easier for people to open up also. So, **if you open up, they open up**.”—Manager 11.

Tot = Total; Jr = Junior consultant; M = Manager.
